# Supplementary figures and images for: Transcriptome profiles of Anopheles gambiae harboring natural low-level Plasmodium infection reveal adaptive advantages for the mosquito
Source: Sci Rep. 2021 Nov 19;11:22578. doi: 10.1038/s41598-021-01842-x (PMC8604914; doi:10.1038/s41598-021-01842-x)

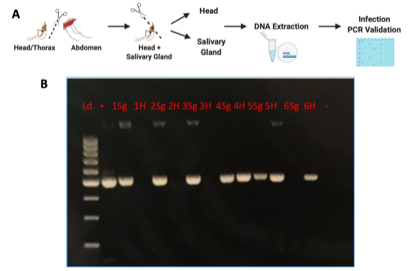

Supplement: Supplementary file 1 — Supplementary Information 1. [file 41598_2021_1842_MOESM1_ESM.tif]
