# Supplementary material for: Transcriptome profiles of Anopheles gambiae harboring natural low-level Plasmodium infection reveal adaptive advantages for the mosquito
Source: Sci Rep. 2021 Nov 19;11:22578. doi: 10.1038/s41598-021-01842-x (PMC8604914; doi:10.1038/s41598-021-01842-x)
Supplement: Supplementary file 3 — Supplementary Information 3. [file 41598_2021_1842_MOESM3_ESM.docx]

**Figure S1.** (A) Schematic representation of gDNA extraction methodology from head and salivary gland tissues of *Plasmodium falciparum* sporozoite infected treatment mosquitoes. (B) Agarose gel (1.5%) electrophoresis image of amplified products using CS2 primer sets. Lanes 1Sg and 1H represent examined salivary gland and head tissues, respectively from treatment *Anopheles. gambiae* s.s. (*An. gambiae*) mosquito 1. Remaining lanes follow a like organization for 5 additional treatment *An. gambiae* mosquitoes with a *P. falciparum* (+) positive and (-) negative control. Lane Ld, 100bp DNA size marker.
